# Characterization of enteropathogenic and Shiga toxin-producing *Escherichia coli* in cattle and deer in a shared agroecosystem

**DOI:** 10.3389/fcimb.2015.00029

**Published:** 2015-04-01

**Authors:** Pallavi Singh, Qiong Sha, David W. Lacher, Jacquelyn Del Valle, Rebekah E. Mosci, Jennifer A. Moore, Kim T. Scribner, Shannon D. Manning

**Affiliations:** ^1^Department of Microbiology and Molecular Genetics, Michigan State UniversityEast Lansing, MI, USA; ^2^Division of Molecular Biology, Center for Food Safety and Applied Nutrition, U.S. Food and Drug AdministrationLaurel, MD, USA; ^3^Biology Department, Grand Valley State UniversityAllendale, MI, USA; ^4^Department of Fisheries and Wildlife, Michigan State UniversityEast Lansing, MI, USA

**Keywords:** STEC, EPEC, EHEC, cattle, deer, transmission, MLST, fingerprinting

## Abstract

Shiga toxin-producing *Escherichia coli* (STEC) is an important foodborne pathogen. Cattle are suggested to be an important reservoir for STEC; however, these pathogens have also been isolated from other livestock and wildlife. In this study we sought to investigate transmission of STEC, enterohemorrhagic *E. coli* (EHEC) and enteropathogenic *E. coli* (EPEC) between cattle and white-tailed deer in a shared agroecosystem. Cattle feces were collected from 100 animals in a Michigan dairy farm in July 2012, while 163 deer fecal samples were collected during two sampling periods (March and June). The locations of deer fecal pellets were recorded via geographic information system mapping and microsatellite multi-locus genotyping was used to link the fecal samples to individual deer at both time points. Following subculture to sorbitol MacConkey agar and STEC CHROMagar, the pathogens were characterized by serotyping, *stx* profiling, and PCR-based fingerprinting; multilocus sequence typing (MLST) was performed on a subset. STEC and EHEC were cultured from 12 to 16% of cattle, respectively, and EPEC was found in 36%. Deer were significantly less likely to have a pathogen in March vs. June where the frequency of STEC, EHEC, and EPEC was 1, 6, and 22%, respectively. PCR fingerprinting and MLST clustered the cattle- and deer-derived strains together in a phylogenetic tree. Two STEC strains recovered from both animal species shared MLST and fingerprinting profiles, thereby providing evidence of interspecies transmission and highlighting the importance of wildlife species in pathogen shedding dynamics and persistence in the environment and cattle herds.

## Introduction

Shiga toxin-producing *Escherichia coli* (STEC) is a leading cause of foodborne infections in the U.S. and represents a major public health concern These pathogens are characterized by their ability to produce Shiga toxin (Stx), which contributes to hemorrhagic colitis and hemolytic uremic syndrome (HUS) in some individuals. Multiple STEC serotypes have been characterized with STEC O157 predominating in the U.S. until recently; serotypes other than O157 (non-O157) have increased in frequency during the last decade (Bettelheim, 2007) and have also contributed to several large-scale outbreaks. One example is the 2011 STEC O104:H4 outbreak in Germany, which infected 3816 people and caused 54 deaths (Frank et al., 2011). In the U.S., it was estimated that up to 168,698 non-O157 infections and 96,534 O157 infections occur each year (Scallan et al., 2011) with most non-O157 infections being caused by the following six serogroups: O26, O103, O111, O121, O45, and O145 (Gould et al., 2013).

The Stx, which is the primary virulence factor of STEC, is encoded by multiple *stx* genes carried on lambdoid bacteriophages (O'brien et al., 1984). A subset of STEC strains also known as enterohemorrhagic *E. coli* (EHEC), possess the locus of enterocyte effacement (LEE) with genes such as *eae* (intimin) that are important for the development of attaching and effacing lesions on intestinal epithelial cells (McDaniel et al., 1995). EHEC O157 was suggested to have evolved from LEE-positive enteropathogenic *E. coli* (EPEC) (Reid et al., 2000), a common cause of infantile diarrhea in developing countries (Kotloff et al., 2013), via the acquisition of Stx-converting bacteriophages. EPEC strains have also been classified into typical EPEC based on the presence of the EPEC adherence factor (EAF) plasmid. This plasmid harbors the *bfp* gene cluster encoding the bundle-forming pilus, which is essential for the initial attachment of EPEC to intestinal epithelial cells (Nataro and Kaper, 1998). Atypical EPEC strains lack *bfp*, though the prevalence of both types may be underestimated in developed countries without routine screening practices in place.

Cattle are considered to be a major reservoir for STEC, EHEC, and EPEC (Beutin et al., 1993; Holland et al., 1999). For STEC, considerable variation has been reported in herd frequencies that range from 0.3 to 56% in beef and 0.2 to 74% in dairy cattle (Hussein and Sakuma, 2005; Hussein, 2007). In addition to cattle, STEC has been recovered from other domesticated animals including sheep, goats, pigs, cats, and dogs (Beutin et al., 1993). Wildlife species including white-tailed deer (*Odocoileus virginianus*) have also been shown to harbor *E. coli* O157 in the U.S., with prevalence estimates of 0.3% of 1608 deer samples in Nebraska (Renter et al., 2001), 0.5% of 609 deer in Georgia (Fischer et al., 2001), and up to 1.8% of 55 deer in Louisiana (Dunn et al., 2004). For non-O157 STEC, recent studies in Germany and Spain have found 83% of roe deer (Mora et al., 2012) and 53% of red and roe deer to be positive (Eggert et al., 2013), while STEC was recovered from 5% of white-tailed deer feces collected in Wisconsin and Minnesota (Ishii et al., 2007). Although these prevalence estimates vary considerably, it is important to note that prevalence is dependent on the type of deer species occupying a given area as well as the type of detection method used; not all studies are comparable. Deer have also been implicated as the STEC source in prior outbreaks including a 2010 O103:H2 outbreak in Minnesota associated with venison (Rounds et al., 2012) and a 2011 O157:H7 outbreak linked to strawberries contaminated by deer feces (Laidler et al., 2013). Collectively, these data suggest that deer are an important reservoir of STEC. Few studies, however, have been conducted in the U.S. to determine the prevalence of STEC, particularly the non-O157 serotypes, among wildlife species, or to estimate transmission frequencies and quantify the genetic diversity of the deer-derived *E. coli* population.

In the farm environment, STEC is commonly transmitted through contact with contaminated cattle feces, water, soil, flies, and birds (Hancock et al., 1998), and only a subset of studies have examined isolates from different deer species and cattle in the same geographic location (Rice et al., 1995; Fischer et al., 2001; Mora et al., 2012). Despite this, it is still not clear how frequently STEC and other diarrheagenic *E. coli* pathotypes are transmitted between cattle and wildlife inhabiting shared landscapes. Consequently, we sought to recover STEC, EHEC, and EPEC from a population of dairy cattle and white-tailed deer (*O. virginianus*) sharing an agroecosystem in Michigan. We examined the pathogen diversity across species and hypothesized that white-tailed deer are an important reservoir for all three *E. coli* pathotypes with interspecies transmission occurring frequently followed by the subsequent diversification in each host.

## Materials and methods

### Field sampling

A total of 163 white-tailed deer fecal samples were collected along pre-selected transects that were established at 100 m intervals in all habitats and pastures across the Kellogg Biological Station property (~18 km^2^) located in southwestern Michigan. Fecal pellet locations were recorded using a hand-held GPS unit. All pellet samples were divided into subsamples for deer genotyping for individual identification and for pathogen screening. The first sampling of deer pellets (*n* = 85) occurred at the end of March 2012 and the second sampling (*n* = 78) took place in June 2012. Fecal pellet locations were entered into a geographic information system (GIS) to examine the spatial distribution of pathogen-positive deer. In addition, fecal grab samples were collected from 100 dairy cattle at the same property in July 2012, 2 weeks following the second deer sampling. All fecal sampling strategies were approved by the Institutional Animal Care and Use Committee at Michigan State University.

### Pathogen isolation

Five grams of feces were inoculated in 2X EC broth (Oxoid Ltd.; Waltham, MA) supplemented with novobiocin (8 mg/L), rifampin (2 mg/L) and potassium tellurite (1 mg/L) for 24 h at 42°C (Jasson et al., 2009), and subcultured to STEC CHROMagar™ (CHROMagar; Paris, France) and sorbitol MacConkey (SMAC) agar. Fecal samples were cultured within a week of collection. Immunomagnetic separation (IMS) using *E. coli* O157 Dynabeads (Invitrogen, Life Technologies; NY, USA) was also used to specifically recover *E. coli* O157 following subculture to STEC CHROM agar™ and overnight growth at 37°C. To maximize our ability to capture the genetic diversity of the *E. coli* population, up to 20 suspect colonies were selected from each plate based on morphological appearance. Colonies were inoculated in Luria-Bertani broth (LB) overnight at 37°C. In all, 1957 suspect *E. coli* colonies were recovered from cattle and 814 colonies were isolated from deer. DNA was extracted and purified using the Qiagen DNA extraction kit (Qiagen Sciences; MD, USA).

### Deer genotyping

Total genomic DNA was extracted from fecal samples collected from white-tailed deer using the Qiagen stool extraction kit. DNA was subjected to genotyping at the following seven nuclear, bi-parentally inherited microsatellite loci: BM1225, BM4107, BM4208, BM6506, CSN3 (Bishop et al., 1994), and RT23, RT27 (Wilson et al., 1997). PCR and genotyping was conducted following protocols described previously (Blanchong et al., 2007, 2008). PCR products were screened using either a Licor Instruments 4200 or Hitachi Instruments FMBIOII scanner. Individuals of known genotype and base pair size standards were run concurrently with all samples on each gel to score the genotypes. All genotypes were scored by two experienced laboratory personnel, while sex was determined using PCR-based methods and sex-linked markers described in a prior study (Lindsay and Belant, 2008). Multi-locus microsatellite profiles were compared across individuals using the GENECAP program (Wilberg and Dreher, 2004) to identify unique and related individuals. Estimates of relatedness among white-tailed deer that were co-infected with enteric pathogens were calculated using maximum likelihood methods as described in a prior study (Wagner et al., 2006). Hypothesized relatedness values (full-siblings, half-siblings, parent-offspring) were evaluated based on likelihood ratio tests.

### Virulence gene profiling and molecular serotyping

The *eae* gene encoding the intimin adhesin and the two most common Stx variant genes, *stx1* and *stx2*, were amplified by multiplex PCR as described (Manning et al., 2008) for suspect *E. coli* colonies. PCR targeting *bfp* (bundle forming pilus) was performed on all *eae*-positive, *stx*-negative isolates as described previously (Trabulsi et al., 2002) for classifying isolates as typical (*bfp*-positive) or atypical (*bfp*-negative) EPEC. For isolates with genome data available, *wzy* (O-antigen polymerase) and *fliC* (flagellar H antigens), which dictate the *E. coli* serotype, were extracted from draft genomes available through the National Center for Biotechnology Information (NCBI) using the Basic Local Alignment Search Tool (Altschul et al., 1990). Sequences were compared to 94 published genomes in NCBI to classify additional serotypes, while primers specific for 14 O- (Table S1) and H- (Table S2) antigens were developed to screen additional isolates lacking genome data.

Three multiplex PCR molecular serotyping assays were developed using malate dehydrogenase (*mdh*) as an internal control (mdh_F41 5′-AGGCGCTTGCACTACTGTTA-3′; mdh_R875 5′- AGCGCGTTCTGTTCAAATG-3′). The assays utilized 2.5 μl of 10 PE buffer, 2.5 μl of 2 mM dNTPs, 2 μl of 25 mM MgCl_2_, 0.5 μl of each primer, 0.3 μl of AmpliTaq Gold (Life Technologies; NY, USA) and 2 μl of DNA template (Table S1). The PCR cycle consisted of an initial denaturation step at 94°C for 10 min followed by 25 cycles of 30 s at 94°C, 10 s at 51°C and 10 s at 72°C, and 1 min at 72°C. All suspect isolates were also screened for the presence of *wzy* specific for serotype O104, which involved denaturation at 95°C for 3 min, followed by 30 cycles of 15 s at 95°C, 15 s at 53°C and 5 s at 72°C. Three separate multiplex PCR targeting the most common (*n* = 14) flagellar H antigens encoded by *fliC* was also performed with *mdh* as internal control. Each multiplex PCR was carried out in a 15-μl reaction using the KAPA2G Fast Multiplex kit (Kapa Biosystems, Inc.; Wilmington, MA) containing 7.5 μl of 2X KAPA2G Fast Multiplex mix, 1 μl of each 10 μM primer and 2 μl of DNA template. The PCR cycle consisted of an initial denaturation at 95°C for 3 min followed by 30 cycles of 30 s at 95°C, 15 s at 55°C and 20 s at 72°C, and final extension of 3 min at 72°C. Isolates that were negative for a known serotype by multiplex PCR and failed to match a published *wzy* or *fliC* sequence were considered non-typeable (NT). Differentiation of NT isolates was determined using RAPD, rep-PCR, and MLST.

### PCR-based DNA fingerprinting

In order to omit duplicate isolates recovered from individual animals and determine transmission frequencies across animals, repetitive PCR (rep-PCR) and random amplified polymorphic DNA (RAPD) PCR were performed on up to 180 isolates using previously described protocols with slight modifications (Hahn et al., 2007; Posse et al., 2007). Both rep-PCR and RAPD have been shown to be useful tools to differentiate closely related STEC strains (Dombek et al., 2000; Mohapatra and Mazumder, 2008). To enhance reproducibility, template DNA concentrations were standardized to100 ng/μl prior to PCR. For RAPD, two primers, 1247 (5′ AAGAGCCCGT 3′) and 1254 (5′ CCGCAGCCAA 3′), were used according to published protocols (Pacheco et al., 1997; Posse et al., 2007). Following gel electrophoresis (1.5%) at 80 V for 2.5 h for rep-PCR and 180 V for 2 h for RAPD-PCR and visualization, gel images were imported in to Bionumerics 5.1 (Applied Maths Inc, TX, USA). Banding patterns were examined using the Dice coefficient and a 0.5% band position tolerance and were compared among suspect colonies isolated from the same animals to omit duplicate strains. Cluster analysis was performed using the unweighted pair group method with arithmetic averages (UPGMA) to identify the total number of strains with distinct banding patterns.

### Multilocus sequence typing (MLST) analysis and genome alignments

A subset of strains found to be distinct by rep-PCR and RAPD profiling were selected for whole genome sequencing using the Illumina MiSeq™ (Illumina Inc. San Diego, CA) platform. Genomes were assembled by Velvet 1.2.07 (Zerbino and Birney, 2008) after trimming with Trimmomatic (Bolger et al., 2014) followed by quality checking with FastQC (http://www.bioinformatics.babraham.ac.uk/projects/fastqc/). Consensus sequences for 15 housekeeping genes (7395 bp) available through the STEC Center at Michigan State University (www.shigatox.net) were used to extract MLST loci from draft genomes using the Basic Local Alignment Search Tool (Altschul et al., 1990). Sequences were aligned and allele and sequence type (ST) assignments were made using the STEC Center database; new allele and ST numbers were assigned to those sequences that were novel and failed to match an existing sequence. All 15 genes were concatenated to construct a neighbor joining tree (p-distance, 1000 bootstrap) in MEGA6 (Tamura et al., 2013) to examine evolutionary relationships between strains. Chi square (χ^2^) and the Fisher's Exact test for sample sizes less than five were used to examine differences in the prevalence of all three *E. coli* pathotypes over time in SAS (version 9.3); a *P* < 0.05 was considered significant and odds ratios (OR) and 95% confidence intervals (95% CI) were calculated to describe the relationships. Contigs of two draft genomes from a deer and cow that were identical by MLST and PCR profiling were ordered individually using Mauve (Darling et al., 2004) and then aligned to each other using progressive Mauve (Darling et al., 2010). Locally collinear blocks, which represent highly conserved regions without rearrangements, were identified using the Mauve rearrangement viewer. Both genomes are publicly available at NCBI (BioSample accession numbers SAMN03402191 and SAMN03402228).

## Results

### STEC, EHEC, and EPEC frequencies in dairy cattle and deer

**Figure 1:**
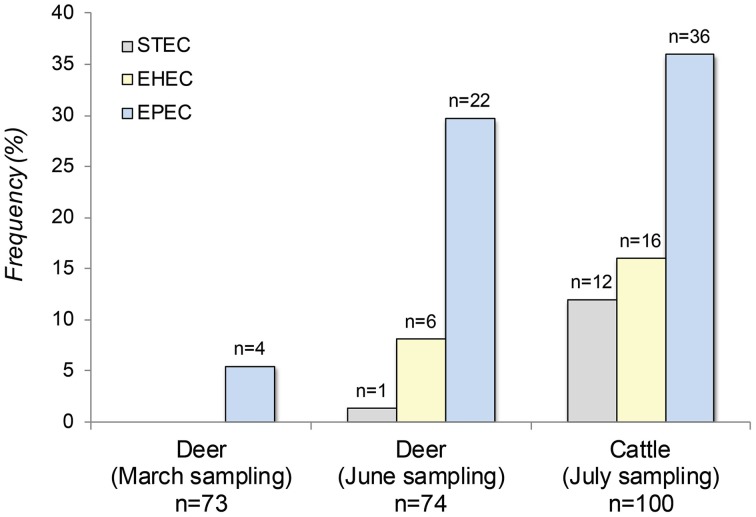
**The frequency of Shiga toxin-producing *E. coli* (STEC), enterohemorrhagic *E. coli* (EHEC) and enteropathogenic *E. coli* (EPEC) in 100 cattle and 147 deer sampled at two time points from the same geographic location**.

Among the 100 cattle sampled in July, 2029 suspect colonies were characterized by PCR for a combination of *stx* and *eae* genes useful for initially classifying the pathogens. EHEC and STEC were recovered from 16 to 12% of cattle, respectively, while 36 (55%) animals had EPEC (Figure 1). The EPEC isolates were further classified as typical (*n* = 4) or atypical (*n* = 32) EPEC based on the presence of *bfp*; two animals were positive for both types of EPEC. Among all 64 pathogen-positive animals, 11 (17%) were positive for both EHEC and EPEC and five (8%) were positive for STEC and EPEC simultaneously.

Among the 163 deer fecal pellets collected at the two samplings, 816 *E. coli* isolates were recovered and characterized by PCR. Deer genotyping data demonstrated that a total of 147 unique animals had been sampled in all. Feces from 73 deer were sampled in March and 74 were sampled in June; 12 of these animals were sampled both in March and in June, while 24 animals were sampled more than once. Notably, the frequency of all three *E. coli* pathotypes increased significantly between March and June (Fishers-Exact test, *P* < 0.0001). During the March sampling, for instance, only four of the 73 (5%) deer were positive for EPEC and neither STEC nor EHEC were detected. In June, EPEC recovery increased significantly to 30% (*n* = 22) in the 74 animals, while STEC and EHEC were found in 1% (*n* = 1) and 8% (*n* = 6) of the deer, respectively. Deer had a greater number of typical EPEC relative to cattle as 14 animals (48%) were positive. In addition, one or more of the three *E. coli* pathotypes were recovered simultaneously from 7% of the deer at the time of the June sampling; five animals positive for EPEC also had either EHEC (*n* = 4) or STEC (*n* = 1). Overall, cattle were significantly more likely to possess any of the three pathogens (OR: 2.7; 95% CI: 1.48, 5.13) as well as EHEC or STEC combined (OR: 3.7; 95% CI: 1.52, 9.09) relative to deer during the June sampling.

### Spatial and temporal distribution of pathogens within the agroecosystem

**Figure 2:**
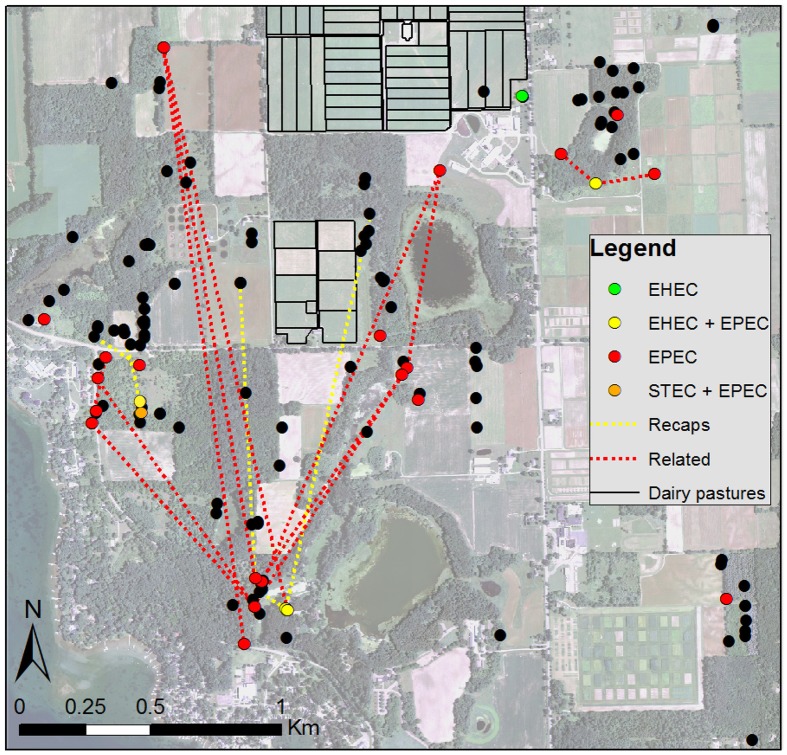
**Aerial photo of the study site showing locations of deer feces (black dots) and feces positive for one of three diarrheagenic *E. coli* pathotypes (colored dots)**. Yellow lines connect fecal groups of the same deer determined from multi-locus genotyping collected during different seasons, including the 12 individual animals that acquired a pathogen over time. Red lines connect fecal groups of genetically related deer (parent-offspring or full siblings) that have moved within the agroecosystem.

Plotting the distribution of deer feces collected over the course of the study identified spatial clustering of feces across the property (Figure 2). The same was true for pathogen-positive fecal samples in that they appeared to cluster together in four specific geographic locations via GIS mapping. These clusters contained 21 of all 33 (64%) pathogen-positive samples. In addition, there was a high probability that deer positive for any of the three pathogens were related to other pathogen-positive deer using the log likelihood ratio test and hypothesized relationships.

Pathogen positivity also changed over the sampling period as five of the 12 deer (42%) sampled in both March and June had acquired at least one of the three pathogens by June. Three of the five animals acquired atypical EPEC as well as either EHEC (*n* = 2) or STEC (*n* = 1) simultaneously, while the two remaining animals acquired typical EPEC (*n* = 1) or both EPEC types (*n* = 1). Importantly, four of the five animals that had acquired a pathogen had fecal samples collected from one of the four pathogen-positive spatial clusters.

### Pathogen characteristics

Because up to 20 suspect isolates were recovered from each of the 93 animals (64 cattle, 29 deer) positive for any of the three *E. coli* pathogens, we sought to examine the characteristics of the bacterial population within and across species. In all, 302 isolates were recovered from 29 deer and 42 cattle and evaluated by PCR for a set of virulence genes. Specifically, 93 pathogen-positive isolates were examined from deer and 209 isolates were examined from cattle. A subset of EHEC and STEC isolates could not be recovered following subculture in both deer (*n* = 3) and cattle (*n* = 15). The *stx* profile was known for the original isolate after culturing directly from the fecal samples; however, upon subculture, the original isolates either lost the Stx-bacteriophage or were no longer cultivable. Because of the latter possibility, the cultivable *stx*-negative *E. coli* isolates were not characterized by serotyping or PCR profiling so as to avoid potential misclassification.

Overall, the *stx* distribution varied among EHEC and STEC isolates recovered from cattle and deer. Only *stx1* was detected in the seven *stx*-positive deer, while *stx1* was detected in 68% (*n* = 19) of the 28 cattle-derived isolates. Isolates with *stx2* and both *stx1* and *stx2* were recovered from 21% (*n* = 6) and 11% (*n* = 3) of the remaining cattle, respectively. Among the EHEC isolates, one had both *stx1* and *stx2*, two had *stx2* only and the remaining 13 isolates had *stx1* alone. The majority of EHEC and STEC isolates were initially classified as non-typeable (NT) by multiplex PCR and thus, additional PCR assays were developed to classify more isolates. Five serotypes were identified from the nine animals with *stx*-positive isolates available including serotypes O103 (*n* = 1), O157:H7 (*n* = 1), O98:H21 (*n* = 5), O169:H16 (*n* = 1), and O53:HNT (*n* = 1); two isolates were ONT:H16. In addition, two of these nine animals had more than one *stx*-positive serotype. Among the *stx*-positive deer isolates available for testing, serotypes O98:H21 (*n* = 1), O103:H2 (*n* = 2) and ONT:H2 (*n* = 1) were identified, while one deer was positive for both of the latter two serotypes.

Among the 26 deer positive for EPEC, serotype O145:H25 predominated and was detected in seven (27%) animals followed by serotypes O177:H11 (*n* = 2), O45:HNT (*n* = 2), O98:H21 (*n* = 1), and O168:H8 (*n* = 1) (Table 1). Most deer (*n* = 17) possessed a NT isolate, while a subset of seven animals had more than one serotype. The cattle-derived EPEC isolates were more diverse. Isolates belonging to O53 predominated in 16 (44%) animals, though the H-antigen varied across O53 isolates (Table 2). The same was true for isolates belonging to O2 (*n* = 4), O115 (*n* = 2), and O169 (*n* = 3). Two additional O:H combinations were also detected as well as a high frequency of isolates (*n* = 38) without a classifiable O-antigen.

**Table 1:** **Diversity of diarrheagenic *E. coli* isolates recovered from 28 deer by PCR profiling**.

**Deer ID**	**Month**	**No. of isolates**	**No. of different isolates**	**Isolates**	**Pattern group**	**Virulence genes**			**Serotype**
						***stx1***	***stx2***	***eae/bfpB***	
2	March	12	2	2s4e	D1			±	NT
				2s6e	D2			±	NT
7	March	5	2	7s7e	D15			+/+	NT
				7s5e	D16			±	NT
33	March	2	1	33s5e	D9			+/+	145:25
37	March	1	1	37s1e	D27			±	145:28
50	June	2	2	50s3e	D9			+/+	145:25
				50SM2e	D3			±	NT
59	June	1	1	59SM1se	D4			±	NT
65	June	3	2	65s1se	D5			±	177:11
				651seM	D6			±	NT:7
66	June	2	2	66s1e1	D7	+		±	98:21
				66s2e	D8			+/+	NT:25
68	June	7	2	68s5eM	D9			+/+	145:25
				68s5eW	D10			±	NT
70	June	1	1	70s1e	D11			±	45:NT
72	June	1	1	72s6e1	D12			±	NT
73	June	1	1	73SM1se1	D13			±	NT
74	June	2	1	74s1se1M	D28	+		±	103:2
75	June	6	1	75s1e	D15			±	NT
76	June	1	1	76s2e	D15			+/+	NT
82	June	1	1	82SM1s1	D17			±	NT
87	June	5	2	87s1se1	D29	+		±	103:2
				87s2e,1	D30	+		±	NT:2
97	June	6	1	97s1e	D15			+/+	NT
98	June	1	1	98s6e	D19			+/+	145:25
100/69	June	9	4	69s5e	D9			±	145:25
				100s1seM	D18			±	98:21
				100s4e	D19			+/+	145:25
101/79	June	2	1	101s5e	D24			+/+	177:11
		1	2	79SM1s1	D14			±	NT
				79-14	D31	+		±	NT
104	June	2	1	104s4eM	D20			±	NT
105	June	3	2	105s2eM	D21			±	NT
				105s2eW	D22			±	45:NT
114	June	1	1	114s1seM	D23			±	168:8
115	June	2	1	115s2eM	D15			+/+	NT
116	June	3	2	116s4e	D9			+/+	145:25
				116SM1se	D25			±	NT:21
121	June	3	2	121s1se	D26			±	NT
				121s6e	D15			+/+	NT
123	June	7	2	123s6e	D9			+/+	145:25
				123s1se	D19			+/+	145:25

**Table 2:** **The diversity of diarrheagenic *E. coli* isolates recovered from 41 cattle by PCR profiling**.

**ID**	**No. of isolate**	**No. of different isolates**	**Isolates**	**Cattle profile**	**Virulence genes**			**Serotype (O:H)**
					***stx1***	***stx2***	***eae*/*bfp***	
778	10	2	778-1	C1			±	NT:10
			778-6M	C8			±	NT:10
779	1	1	779-1	C10			±	53:10
780	1	1	780-1	C10			±	53:NT
789	1	1	789-1	C2			±	53:NT
790	2	1	790-S1	C12			±	NT
791	1	1	791-1	C9	+	+	+	157:7
792	1	1	792-Sw1	C6			±	2:25
800	2	1	800-1M	C13			+/+	53:10
802	1	1	802-1	C4			±	53:10
805	1	1	805-1M	C4			±	NT
809	5	2	809-1	C18	+		+	98:21
			809-2	C16			±	115:25
811	1	1	811-Sw1	C14			±	169:16
812	3	3	812-2	C19			±	169:16
			812-3W	C20			±	53:21
			812-1	C6			±	2:25
813	1	1	813-1	C10			±	53:38
820	1	1	820-Sw1	C21			±	NT:2
821	1	1	821-2	C3			±	NT:10
823	3	2	823-2	C22			±	115:10
			823-3M	C35			±	2:21
824	2	1	824-Sm10	C44		+	−	NT:16
825	5	3	825-1M	C18	+		+	98:21
			825-4M	C17	+		+	53:NT
			825-1W	C23			±	53:10
826	14	3	826-1	C4			±	NT
			826-5M	C24			+/+	177:11
			826-10M	C10			±	53:38
827	1	1	827-1M	C25			±	169:8
828	8	1	828-Sm3	C43	+		+	169:16
829	4	2	829-1M	C30			+/+	53:21
			829-6M	C10			±	53:NT
830	14	3	830-16M	C27			±	NT:21
			830-11	C26			±	NT:38
			830-7M	C28			±	53:11
834	16	1	834-Sm4	C44		+	−	NT:16
835	3	2	835-1M	C10			±	53:10
			835-7W	C29			±	53:21
837	20	4	837-10M	C18	+		+	98:21
			837-14M	C31			±	103:2
			837-13M	C1			±	NT:10
			837-16W	C32			±	NT:21
838	7	1	838-1M	C24			±	177:11
845	1	1	845-1M	C34			+/+	NT:21
848	1	1	848-1M	C1			±	NT:10
849	2	2	849-3M	C5			±	2:25
			849-2M	C10			±	53:38
850	7	1	850-1M	C18	+		+	98:21
858	14	3	858-10M	C7			±	NT:21
			858-11M	C36			±	NT:21
			858-12W	C12			±	53:4
860	4	2	860-3M	C33			±	103:NT
			860-1M	C1			±	NT:10
861	4	2	861-1M	C11			±	53:10
			861-6M	C45			±	53:7
863	2	2	863-2M	C38			±	6:10
			863-1M	C37			±	NT:21
866	14	2	866-10M	C39			±	NT:21
			866-2M	C7			±	NT:25
870	1	1	870-1M	C40			±	NT:10
871	18	2	871-10M	C41			±	103:NT
			871-11M	C1			±	NT:10
872	3	1	872-3M	C1			±	NT:10
873	8	2	873-1M	C18	+		+	98:21
			873-4SW	C42		+	+	91:21

*Only those isolates negative for stx were examined for bfp. Cattle profiles represent an arbitrary number assigned to each unique combination of banding patterns as determined by RAPD and REP-PCR*.

### Pathogen diversity

RAPD and rep-PCR were utilized to exclude duplicate isolates from each animal and identify transmission events and candidates for MLST. Among the 302 isolates from cattle and deer, 75 distinct DNA fingerprint profiles were identified. A total of 44 profiles were identified in the cattle and 31 profiles were identified in the deer. In all, 27 cattle had >1 isolate characterized by PCR profiling and 18 animals (28%) had >1 fingerprint profile with an average of two profiles per animal and a maximum of four (Table 2). Most isolates with unique PCR profiles from individual animals (*n* = 13) were distinct by serotyping. Only two animals had isolates with distinct PCR profiles and identical serotypes, though two additional animals had distinct profiles with identical O-antigens but not H-antigens. On the contrary, more than one isolate was characterized from 19 deer and 13 of these animals (45%) had >1 profile with an average of one and maximum of four profiles per animal (Table 1). Among the serotyped deer isolates from individual animals with distinct PCR profiles, most (*n* = 8) were also different by serotyping. A subset of isolates from two animals, however, shared the same serotype despite having distinct PCR profiles.

Isolates of the same *E. coli* pathotype were not always identical by PCR profiling unless they represented the same serotype. For cattle, five distinct fingerprint patterns were identified among the nine EHEC isolates, whereas the two STEC isolates shared one pattern. All five deer-derived EHEC isolates, however, had distinct profiles even though two of the five isolates had identical serotypes. The EPEC isolates were considerably more diverse in both species. Among the EPEC isolates from cattle, 38 unique patterns were identified, whereas 26 unique patterns were found in deer. Multiple fingerprint profiles were also identified for each of the typical EPEC isolates recovered from cattle (*n* = 4), while the 15 typical EPEC strains from deer only grouped into five different PCR profiles.

### Intraspecies and interspecies transmission

As determined by PCR profiling, a subset of isolates recovered from the cattle and deer populations were shared within and/or between species. Indeed, a cluster analysis based on the rep-PCR profiles demonstrated that the isolates recovered from both cattle and deer were not genetically distinct as isolates from both hosts were found on similar branches of the phylogenetic tree (Figure S1). Specifically, nine fingerprint patterns were identified across multiple cattle and three patterns were found in more than one deer. Seven cattle with O53 EPEC isolates, for example, were classified as belonging to cattle PCR profile pattern 10 (C10), while six animals had ONT:10 isolates representing pattern 1 (C1). PCR profile 18 (C18) was also detected in five different cattle, while the remaining six PCR patterns were found in two or three animals (Table 2). For the deer, two PCR patterns represented the EPEC O145:H25 isolates, which were found in nine different animals, while six animals had ONT isolates with identical PCR profiles (Table 1).

There was evidence of EHEC and STEC transmission within cattle. Each of the *stx1*-positive EHEC O98:H21 isolates recovered from five different cattle, for example, had identical fingerprint profiles as did the two STEC ONT:H16 isolates. The cattle-derived EHEC NT, O91:H21 and O169:H16 isolates, however, were each found in only one animal as were the five deer-derived EHEC isolates.

**Figure 3:**
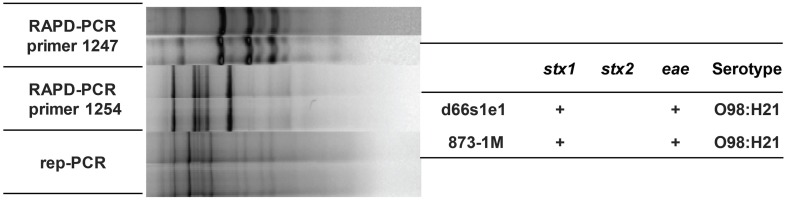
**Isolates recovered from one deer (d66s1se) and one cow (873-1M) had identical fingerprint patterns, serotypes and virulence gene profiles, thereby providing evidence for interspecies transmission**.

Furthermore, three of the four EHEC serotypes (O98:H21, O103:H2, and O177:H11) from deer were also present in the cattle herd. The EPEC O103:H2 isolates from cattle had distinct fingerprint profiles when compared to the deer-derived EHEC O103 isolates as did the O177:H11 isolates. On the contrary, the EHEC O98:H21 isolate recovered from a deer had a fingerprint profile that matched several cattle-derived isolates of the same serotype by all three PCR profiling methods (Figure 3). These isolates and a subset of additional isolates from 17 cattle and 7 deer were examined by MLST to better understand the degree of relatedness between isolates from both species. Matching fingerprint profiles were not observed for those isolates recovered from the 12 deer sampled in both March and June, indicating that new strains were acquired over time. Notably, one of the 12 deer acquired two distinct EPEC isolates that were also recovered from five different animals during the June sampling.

### Evolutionary relationships between deer- and cattle-derived isolates

**Figure 4:**
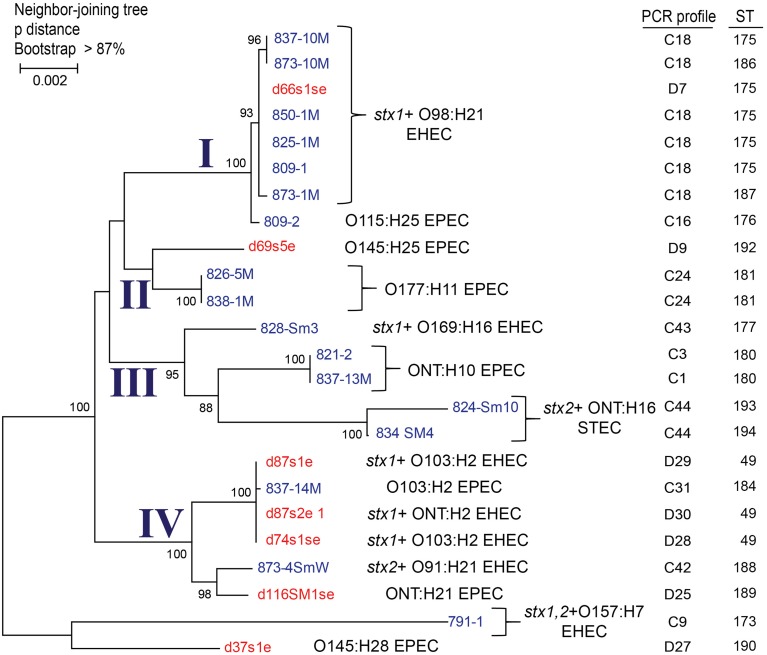
**A phylogenetic tree constructed using 15 multilocus sequence typing loci in 24 pathogenic *E. coli* isolates constructed using the Neighbor-joining method with 1000 bootstrap replications**. The evolutionary distances were calculated using p-distance and represent the number of base differences per site. Clusters I–IV were identified based on >95% bootstrap support and serotypes, virulence gene profiles, and sequence types (STs) are noted. Deer isolates are indicated in red and cattle isolates are in blue. PCR profiles represent an arbitrary number that was assigned to each unique combination of PCR profiles per animal.

**Figure 5:**
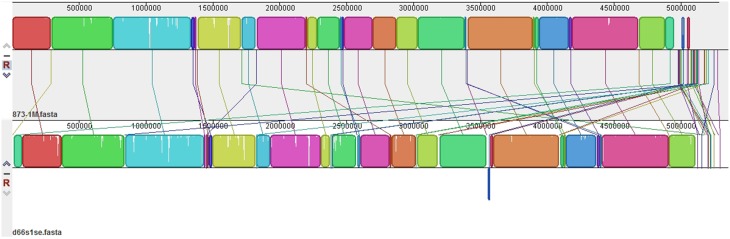
**Alignment of two *stx1*-positive EHEC O98:H21 genomes from a cow (top) and deer (bottom) that were identical by PCR profiling and multilocus sequence typing**. Identical colored boxes are pairwise locally collinear blocks, which represent regions of highly conserved sequences without rearrangements, as determined used progressive Mauve (Darling et al., 2010). White spaces are indicative of regions with low sequence coverage, while the lines represent regions that match in each genome.

Application of MLST targeting 15 housekeeping genes (*n* = 7473 nucleotides) resulted in a phylogenetic tree with deer and cattle isolates grouping together (Figure 4). A total of four clusters were identified with two being specific to cattle and none specific to deer. The remaining two clusters contained both cattle- and deer-derived isolates. Overlaying the PCR profiling data on to the phylogenetic tree demonstrated that many isolates with identical PCR profiles clustered together. In most cases, isolates with the same sequence type (ST) also grouped together. The cattle-specific cluster II, for instance, consisted of O177:H11 EPEC isolates from two animals with identical PCR profiles and STs, while cluster III contained matching ONT:H10 isolates from two different animals as well as three closely related isolates with different serotypes, *stx* and PCR profiles, and STs. The same was true for isolates comprising cluster IV as they varied by *stx* profile, serotype, ST, and PCR profile, though they were not host specific and were recovered from both deer and cattle. Specifically, the two groups of isolates comprising cluster IV had slightly different characteristics including the lack of *stx1, stx2*, or a NT O-antigen, which could have resulted in distinct banding patterns by PCR profiling. Three of the four deer-derived strains from cluster IV, however, had the same genetic backbone (ST-49) that differed from the cattle strains comprising this cluster. Notably, two ST-49 isolates (TW07697 and TW08101), available within the STEC Center repository, were previously recovered from humans with diarrhea and bloody diarrhea. Among all four clusters, the most closely related and broadly distributed isolates belonged to cluster I. This cluster included highly similar isolates from different cattle and one deer (d66s1se). Although all of the O98:H21 cluster I isolates had *stx1* and were identical by PCR profiling, only five isolates had the same ST. Consequently, two representative cluster I isolates recovered from both species were sequenced. Genome alignments demonstrated that the deer- and cattle-derived strains from cluster I are highly similar (Figure 5) and suggests that transmission between species occurred previously.

## Discussion

Given the worldwide burden of diarrheagenic *E. coli* in human infections, it is important to know the distribution of various pathotypes in reservoir species such as cattle (Beutin et al., 1993; Holland et al., 1999). Knowledge of the diversity and genetic relatedness of diarrheagenic *E. coli* in external reservoirs is also important to determine whether certain strain types are more likely to be transmitted within species and whether there is evidence of interspecies transmission. In this study, we observed a high frequency (64%) of STEC, EHEC, and EPEC in dairy cattle from one Michigan herd as well as in white-tailed deer (40%) sharing an agroecosystem. Although cattle were significantly more likely to harbor all three *E. coli* pathotypes than deer, though STEC and EHEC were detected in 9% of deer feces. It is difficult, however, to determine how pathogen prevalence compares to estimates from other geographic locations (Ishii et al., 2007; Sasaki et al., 2013) as different deer species, wildlife-livestock interactions, and culture methods can significantly impact prevalence and detection. For instance, culturing samples with potassium tellurite will enhance recovery of tellurite-resistant EHEC as was demonstrated previously (Tzschoppe et al., 2012), and may have resulted in a less diverse pathogen population. Nevertheless, high frequencies of genetically diverse *E. coli* isolates were still recovered from deer and cattle in this study. Because many of these isolates possessed virulence characteristics that have previously been correlated with more severe clinical infections in humans, it is clear that both deer and cattle are important reservoirs of pathogenic STEC and EHEC in Michigan.

Among the *stx*-positive isolates recovered from the deer and cattle in this study, most (63%) were classified as EHEC, which possesses the LEE and was suggested to be more virulent in humans (Nataro and Kaper, 1998). Similarly, a high frequency of cattle-derived STEC and EHEC isolates were positive for the Stx2-encoding phage, which has been linked to more severe disease when compared to isolates containing other Stx phages (Persson et al., 2007). The molecular serotyping assays also identified a subset of deer- and cattle-derived isolates with serotypes that have previously been linked to disease in human patients. One EHEC isolate belonging to serotype O157 was detected in a cow, while the remainder of STEC or EHEC isolates from both deer and cattle belonged to non-O157 serotypes. This high frequency of non-O157 serotypes mimics the increasing frequency of non-O157 serotypes in human infections (Bettelheim, 2007) and has contributed to an enhanced awareness of their presence in foods (Gould et al., 2013). Even though hundreds of serotypes have been described, serotypes O157, O111, O145, O45, O103, O121, and O26 were previously suggested to be the most important for human health (Brooks et al., 2005). In both the deer and cattle sampled through this study, we recovered four of these seven serotypes, although a subset of the isolates were classified as EPEC and lacked genes encoding the Stx.

Overall, high frequencies of EPEC representing 18 and seven different serotypes were detected in the cattle and deer, respectively; serotypes could not be determined for 44% of the isolates. These strain types may represent emerging serotypes in both reservoirs, however, it is not clear whether they have the ability to be transmitted to humans and cause disease. It is likely that a subset of the isolates are ruminant-specific, with only a proportion having the ability to infect humans. Additional studies are therefore needed to determine which deer- and cattle-derived isolates exhibit similar properties *in vitro* as human-derived isolates. EPEC strains are important not only because they can cause diarrhea in humans, but also because they can be instantaneously converted into EHEC following infection with a Stx bacteriophage, which enhances its virulence. As proof of concept, a recent study demonstrated that multiple Stx2-bacteriophages were capable of infecting all types of pathogenic *E. coli*, including EPEC (Tozzoli et al., 2014). Although Stx phage infection is critical for the emergence of novel pathogens, some *E. coli* hosts and Stx-phage combinations are likely to result in a more stable pathogen that can survive in external reservoirs and be transmitted to humans. The *stx2*-positive *E. coli* O104:H4 German outbreak strain represents a good example (Frank et al., 2011). In this study, we initially recovered multiple STEC and EHEC isolates that became negative for Stx-phages following subculture, thereby providing support for this hypothesis. These isolates may represent a population of bacteria that survive better in ruminants. Future work should focus on detecting and differentiating stable reservoir-derived *E. coli* pathogens from less stable pathogens in order to identify bacterial- and phage-specific characteristics that may useful for predicting which strains are most pathogenic and could be targeted for detection in foods.

Because we selected up to 20 isolated colonies per animal, we were also able to investigate the diversity of the EHEC, STEC, and EPEC populations in both cattle and deer living in close proximity. DNA fingerprinting techniques such as PFGE have previously been used to study the transmission of STEC isolates in cattle (Rice et al., 1995; Fischer et al., 2001; Mora et al., 2012). A Texas study, for instance, found STEC O157:H7 isolates from cattle and deer sampled at the same location to have identical PFGE patterns (Rice et al., 1995). Another prior study in the southeastern U.S. failed to identify shared STEC O157:H7 strains across the two species, however, the prevalence of STEC was low and sample collection took place during different parts of the year (Fischer et al., 2001). Although PFGE is the gold standard, limitations associated with cost and time can prevent its use in molecular epidemiological studies, particularly when duplicate isolates are expected. Here, we utilized a combination of three PCR-based profiling methods to omit duplicate isolates within each animal and identify candidates for serotyping and MLST, which is more amenable to phylogenetic analyses. The application of repetitive and RAPD PCR profiling has demonstrated a diverse pathogen population in both deer and cattle with isolates from both species clustering together in a dendrogram using UPGMA. For the most part, we observed identical PCR profiles among isolates with the same serotype, though there were exceptions. These data are consistent with results from a prior study of roe deer and cattle located in the same area in Spain as STEC strains of multiple serotypes varied by PFGE despite being closely related (Mora et al., 2012). Although these data suggest that serotyping is less useful for characterizing isolates, we suggest the use of serotyping to initially determine which isolates are different. Additional methods are needed to assess whether isolates with identical serotypes are genetically distinct.

Since none of the deer sampled in our Michigan study were positive for STEC or EHEC in March vs. June, we expect that the timing of sample collection is critical for pathogen detection in reservoir species as are the geographic location and landscape features. These findings are consistent with other studies that have observed higher STEC and EHEC frequencies in the warmer summer months relative to the colder winter months (Chapman et al., 1997; Cobbold et al., 2004). Moreover, we observed that the pathogen-positive deer samples clustered spatially by GIS mapping (Figure 2) It is therefore possible that these pathogens persist in specific environments, particularly in mild to warm temperatures that support growth, and that transmission occurs from environmental sources to both wildlife and livestock as well as between animals occupying the same niche. Given that deer travel great distances within a given ecosystem, deer movements are also likely to be important for pathogen transmission, particularly to livestock located nearby. Collectively, these data raise the question about the role that specific environments or wildlife species play in the maintenance of existing pathogen populations as well as the emergence of novel pathogens. Future studies are therefore warranted to better understand these interactions as well as identify factors important for pathogen persistence across environments and reservoir species.

Similar to the PCR-based profiling data generated in this study, the application of MLST to a subset of isolates demonstrated that cattle- and deer-derived STEC, EHEC, and EPEC are closely related. Isolates recovered from both species were integrated within the phylogeny, suggesting that diversification within each host occurs following pathogen transmission and adaptation. The MLST analysis of a subset of isolates identified four phylogenetic clusters; two of these clusters contained isolates that were specific only to cattle (Figure 4). In most cases, the PCR profiles were identical for isolates that grouped together by MLST; however, several exceptions were observed, which could be due to genetic variation outside of the MLST loci or inaccurate PCR profiles. Indeed, PCR-profiling is not an ideal strategy for assessing genetic relatedness unless it is paired with a sequenced based method such as MLST. In this study, the combination of both PCR profiling and MLST enhanced our ability to identify unique isolates circulating in both deer and cattle and to subsequently determine how related they were to each other. Using this approach, we recovered *stx1*-positive O98 EHEC isolates from several cattle and one deer that were highly similar at the genomic level, thereby providing additional support for EHEC transmission across reservoir species. Nonetheless, a more complete genomic analysis is needed to pinpoint specific differences across closely related isolates as is a longitudinal study to determine the direction of transmission and identify risk factors including landscape features and wildlife densities that can impact pathogen prevalence in reservoir species.

## Author contributions

JD, JM, RM, and KS collected samples; PS, QS, JD, RM, DL, and JM designed assays and generated data; QS, PS, JM, KS, and SM analyzed data; SM and KS designed the study; and PS, QS, KS, and SM drafted the manuscript.

### Conflict of interest statement

The authors declare that the research was conducted in the absence of any commercial or financial relationships that could be construed as a potential conflict of interest.

## References

[B1] Altschul S. F. Gish W. Miller W. Myers E. W. Lipman D. J. (1990). Basic local alignment search tool. J. Mol. Biol. 215, 403–410. 10.1016/S0022-2836(05)80360-2 2231712

[B2] Bettelheim K. A. (2007). The non-O157 shiga-toxigenic (verocytotoxigenic) *Escherichia coli*; under-rated pathogens. Crit. Rev. Microbiol. 33, 67–87. 10.1080/10408410601172172 17453930

[B3] Beutin L. Geier D. Steinruck H. Zimmermann S. Scheutz F. (1993). Prevalence and some properties of verotoxin (Shiga-like toxin)-producing *Escherichia coli* in 7 different species of healthy domestic animals. J. Clin. Microbiol. 31, 2483–2488. 8408571 10.1128/jcm.31.9.2483-2488.1993 PMC265781

[B4] Bishop M. D. Kappes S. M. Keele J. W. Stone R. T. Sunden S. L. Hawkins G. A.. (1994). A genetic linkage map for cattle. Genetics 136, 619–639. 7908653 10.1093/genetics/136.2.619 PMC1205813

[B5] Blanchong J. A. Samuel M. D. Scribner K. T. Weckworth B. Lagenberg J. Filcek K. (2008). Landscape genetics and the spatial distribution of chronic wasting disease. Biol. Lett. 4, 130–133. 10.1098/rsbl.2007.0523 18077240 PMC2412942

[B6] Blanchong J. A. Scribner K. T. Kravchenko A. N. Winterstein S. R. (2007). A genealogical basis for disease infection risk in free-ranging wildlife. Biol. Lett. 2, 103–107.17443977

[B7] Bolger A. M. Lohse M. Usadel B. (2014). Trimmomatic: a flexible trimmer for Illumina sequence data. Bioinformatics 30, 2114–2120. 10.1093/bioinformatics/btu170 24695404 PMC4103590

[B8] Brooks J. T. Sowers E. G. Wells J. G. Greene K. D. Griffin P. M. Hoekstra R. M.. (2005). Non-O157 Shiga toxin-producing *Escherichia coli* infections in the United States, 1983-2002. J. Infect. Dis. 192, 1422–1429. 10.1086/466536 16170761

[B9] Chapman P. A. Siddons C. A. Gerdan Malo A. T. Harkin M. A. (1997). A 1-year study of *Escherichia coli* O157 in cattle, sheep, pigs and poultry. Epidemiol. Infect. 119, 245–250. 10.1017/S0950268897007826 9363024 PMC2808847

[B10] Cobbold R. N. Rice D. H. Szymanski M. Call D. R. Hancock D. D. (2004). Comparison of shiga-toxigenic *Escherichia coli* prevalences among dairy, feedlot, and cow-calf herds in Washington State. Appl. Environ. Microbiol. 70, 4375–4378. 10.1128/AEM.70.7.4375-4378.2004 15240323 PMC444803

[B11] Darling A. C. Mau B. Blattner F. R. Perna N. T. (2004). Mauve: multiple alignment of conserved genomic sequence with rearrangements. Genome Res. 14, 1394–1403. 10.1101/gr.2289704 15231754 PMC442156

[B12] Darling A. E. Mau B. Perna N. T. (2010). progressiveMauve: multiple genome alignment with gene gain, loss and rearrangement. PLoS ONE 5:e11147. 10.1371/journal.pone.0011147 20593022 PMC2892488

[B13] Dombek P. E. Johnson L. K. Zimmerley S. T. Sadowsky M. J. (2000). Use of repetitive DNA sequences and the PCR to differentiate *Escherichia coli* isolates from human and animal sources. Appl. Environ. Microbiol. 66, 2572–2577. 10.1128/AEM.66.6.2572-2577.2000 10831440 PMC110583

[B14] Dunn J. R. Keen J. E. Moreland D. Alex T. (2004). Prevalence of *Escherichia coli* O157:H7 in white-tailed deer from Louisiana. J. Wildl. Dis. 40, 361–365. 10.7589/0090-3558-40.2.361 15362843

[B15] Eggert M. Stuber E. Heurich M. Fredriksson-Ahomaa M. Burgos Y. Beutin L.. (2013). Detection and characterization of Shiga toxin-producing *Escherichia coli* in faeces and lymphatic tissue of free-ranging deer. Epidemiol. Infect. 141, 251–259. 10.1017/S0950268812000246 22370185 PMC9152051

[B16] Fischer J. R. Zhao T. Doyle M. P. Goldberg M. R. Brown C. A. Sewell C. T.. (2001). Experimental and field studies of *Escherichia coli* O157:H7 in white-tailed deer. Appl. Environ. Microbiol. 67, 1218–1224. 10.1128/AEM.67.3.1218-1224.2001 11229913 PMC92716

[B17] Frank C. Werber D. Cramer J. P. Askar M. Faber M. An Der Heiden M.. (2011). Epidemic profile of Shiga-toxin-producing *Escherichia coli* O104:H4 outbreak in Germany. N. Engl. J. Med. 365, 1771–1780. 10.1056/NEJMoa1106483 21696328

[B18] Gould L. H. Mody R. K. Ong K. L. Clogher P. Cronquist A. B. Garman K. N.. (2013). Increased recognition of non-O157 Shiga toxin-producing *Escherichia coli* infections in the United States during 2000-2010: epidemiologic features and comparison with *E. coli* O157 infections. Foodborne. Pathog. Dis. 10, 453–460. 10.1089/fpd.2012.1401 23560425

[B19] Hahn D. Gaertner J. Forstner M. R. J. Rose F. L. (2007). High-resolution analysis of salmonellae from turtles within a headwater spring ecosystem. FEMS Microbiol. Ecol. 60, 148–155. 10.1111/j.1574-6941.2007.00275.x 17250751

[B20] Hancock D. D. Besser T. E. Rice D. H. Ebel E. D. Herriott D. E. Carpenter L. V. (1998). Multiple sources of *Escherichia coli* O157 in feedlots and dairy farms in the northwestern USA. Prev. Vet. Med. 35, 11–19. 10.1016/S0167-5877(98)00050-6 9638776

[B21] Holland R. E. Wilson R. A. Holland M. S. Yuzbasiyan-Gurkan V. Mullaney T. P. White D. G. (1999). Characterization of eae+ *Escherichia coli* isolated from healthy and diarrheic calves. Vet. Microbiol. 66, 251–263. 10.1016/S0378-1135(99)00013-9 10384886 PMC7117348

[B22] Hussein H. S. (2007). Prevalence and pathogenicity of Shiga toxin-producing *Escherichia coli* in beef cattle and their products. J. Anim. Sci. 85, E63–E72. 10.2527/jas.2006-421 17060419

[B23] Hussein H. S. Sakuma T. (2005). Invited review: prevalence of Shiga toxin-producing *Escherichia coli* in dairy cattle and their products. J. Dairy Sci. 88, 450–465. 10.3168/jds.S0022-0302(05)72706-5 15653509

[B24] Ishii S. Meyer K. P. Sadowsky M. J. (2007). Relationship between phylogenetic groups, genotypic clusters, and virulence gene profiles of *Escherichia coli* strains from diverse human and animal sources. Appl. Environ. Microbiol. 73, 5703–5710. 10.1128/AEM.00275-07 17644637 PMC2074926

[B25] Jasson V. Rajkovic A. Baert L. Debevere J. Uyttendaele M. (2009). Comparison of enrichment conditions for rapid detection of low numbers of sublethally injured *Escherichia coli* O157 in food. J. Food Prot. 72, 1862–1868. 19777887 10.4315/0362-028x-72.9.1862

[B26] Kotloff K. L. Nataro J. P. Blackwelder W. C. Nasrin D. Farag T. H. Panchalingam S.. (2013). Burden and aetiology of diarrhoeal disease in infants and young children in developing countries (the Global Enteric Multicenter Study, GEMS): a prospective, case-control study. Lancet 382, 209–222. 10.1016/S0140-6736(13)60844-2 23680352

[B27] Laidler M. R. Tourdjman M. Buser G. L. Hostetler T. Repp K. K. Leman R.. (2013). *Escherichia coli* O157:H7 infections associated with consumption of locally grown strawberries contaminated by deer. Clin. Infect. Dis. 57, 1129–1134. 10.1093/cid/cit468 23876397

[B28] Lindsay A. R. Belant J. L. (2008). A simple and improved PCR-based technique for white-tailed deer (*Odocoileus virginianus*) sex identification. Conserv. Genet. 9, 443–447 10.1007/s10592-007-9326-y

[B29] Manning S. D. Motiwala A. S. Springman A. C. Qi W. Lacher D. W. Ouellette L. M.. (2008). Variation in virulence among clades of *Escherichia coli* O157:H7 associated with disease outbreaks. Proc. Natl. Acad. Sci. U.S.A. 105, 4868–4873. 10.1073/pnas.0710834105 18332430 PMC2290780

[B30] McDaniel T. K. Jarvis K. G. Donnenberg M. S. Kaper J. B. (1995). A genetic locus of enterocyte effacement conserved among diverse enterobacterial pathogens. Proc. Natl. Acad. Sci. U.S.A. 92, 1664–1668. 10.1073/pnas.92.5.1664 7878036 PMC42580

[B31] Mohapatra B. R. Mazumder A. (2008). Comparative efficacy of five different rep-PCR methods to discriminate *Escherichia coli* populations in aquatic environments. Water Sci. Technol. 58, 537–547. 10.2166/wst.2008.424 18725719

[B32] Mora A. Lopez C. Dhabi G. Lopez-Beceiro A. M. Fidalgo L. E. Diaz E. A.. (2012). Seropathotypes, phylogroups, *stx* subtypes, and intimin types of wildlife-carried, Shiga toxin-producing *Escherichia coli* strains with the same characteristics as human-pathogenic isolates. Appl. Environ. Microbiol. 78, 2578–2585. 10.1128/AEM.07520-11 22307301 PMC3318799

[B33] Nataro J. P. Kaper J. B. (1998). Diarrheagenic *Escherichia coli*. Clin. Microbiol. Rev. 11, 142–201. 9457432 10.1128/cmr.11.1.142 PMC121379

[B34] O'brien A. D. Newland J. W. Miller S. F. Holmes R. K. Smith H. W. Formal S. B. (1984). Shiga-like toxin-converting phages from *Escherichia coli* strains that cause hemorrhagic colitis or infantile diarrhea. Science 226, 694–696 10.1126/science.6387911 6387911

[B35] Pacheco A. B. F. Guth B. E. C. Soares K. C. C. Nishimura L. Dealmeida D. F. Ferreira L. C. S. (1997). Random amplification of polymorphic DNA reveals serotype-specific clonal clusters among enterotoxigenic *Escherichia coli* strains isolated from humans. J. Clin. Microbiol. 35, 1521–1525. 9163473 10.1128/jcm.35.6.1521-1525.1997 PMC229778

[B36] Persson S. Olsen K. E. Ethelberg S. Scheutz F. (2007). Subtyping method for *Escherichia coli* shiga toxin (verocytotoxin) 2 variants and correlations to clinical manifestations. J. Clin. Microbiol. 45, 2020–2024. 10.1128/JCM.02591-06 17446326 PMC1933035

[B37] Posse B. De Zutter L. Heyndrickx M. Herman L. (2007). Metabolic and genetic profiling of clinical O157 and non-O157 Shiga-toxin-producing *Escherichia coli*. Res. Microbiol. 158, 591–599. 10.1016/j.resmic.2007.06.001 17845842

[B38] Reid S. D. Herbelin C. J. Bumbaugh A. C. Selander R. K. Whittam T. S. (2000). Parallel evolution of virulence in pathogenic *Escherichia coli*. Nature 406, 64–67. 10.1038/35017546 10894541

[B39] Renter D. G. Sargeant J. M. Hygnstorm S. E. Hoffman J. D. Gillespie J. R. (2001). *Escherichia coli* O157:H7 in free-ranging deer in Nebraska. J. Wildl. Dis. 37, 755–760. 10.7589/0090-3558-37.4.755 11763739

[B40] Rice D. H. Hancock D. D. Besser T. E. (1995). Verotoxigenic *Escherichia coli* O157 colonization of wild deer and range cattle. Vet. Rec. 137, 524–524. 10.1136/vr.137.20.524 8588285

[B41] Rounds J. M. Rigdon C. E. Muhl L. J. Forstner M. Danzeisen G. T. Koziol B. S.. (2012). Non-O157 Shiga Toxin-producing *Escherichia coli* associated with venison. Emerg. Infect. Dis. 18, 279–282. 10.3201/eid1802.110855 22305114 PMC3310449

[B42] Sasaki Y. Goshima T. Mori T. Murakami M. Haruna M. Ito K.. (2013). Prevalence and antimicrobial susceptibility of foodborne bacteria in wild boars (*Sus scrofa*) and wild deer (*Cervus nippon*) in Japan. Foodborne Pathog. Dis. 10, 985–991. 10.1089/fpd.2013.1548 24161070

[B43] Scallan E. Hoekstra R. M. Angulo F. J. Tauxe R. V. Widdowson M. A. Roy S. L.. (2011). Foodborne illness acquired in the United States–major pathogens. Emerg. Infect. Dis. 17, 7–15. 10.3201/eid1701.P11101 21192848 PMC3375761

[B44] Tamura K. Stecher G. Peterson D. Filipski A. Kumar S. (2013). MEGA6: molecular evolutionary genetics analysis version 6.0. Mol. Biol. Evol. 30, 2725–2729. 10.1093/molbev/mst197 24132122 PMC3840312

[B45] Tozzoli R. Grande L. Michelacci V. Ranieri P. Maugliani A. Caprioli A.. (2014). Shiga toxin-converting phages and the emergence of new pathogenic *Escherichia coli*: a world in motion. Front. Cell. Infect. Microbiol. 4:80. 10.3389/fcimb.2014.00080 24999453 PMC4064290

[B46] Trabulsi L. R. Keller R. Gomes T. A. T. (2002). Typical and atypical enteropathogenic *Escherichia coli*. Emerg. Infect. Dis. 8, 508–513. 10.3201/eid0805.010385 11996687 PMC2732489

[B47] Tzschoppe M. Martin A. Beutin L. (2012). A rapid procedure for the detection and isolation of enterohaemorrhagic *Escherichia coli* (EHEC) serogroup O26, O103, O111, O118, O121, O145 and O157 strains and the aggregative EHEC O104:H4 strain from ready-to-eat vegetables. Int. J. Food Microbiol. 152, 19–30. 10.1016/j.ijfoodmicro.2011.10.009 22071287

[B48] Wagner A. P. Creel S. Kalinowski S. T. (2006). Estimating relatedness and relationships using microsatellite loci with null alleles. Heredity (Edinb) 97, 336–345. 10.1038/sj.hdy.6800865 16868566

[B49] Wilberg M. J. Dreher B. P. (2004). GENECAP: a program for analysis of nuclear data for capture-recapture population estimation. Mol. Ecol. Notes 4, 783–785 10.1111/j.1471-8286.2004.00797.x

[B50] Wilson G. A. Strobeck C. Wu L. Coffin J. W. (1997). Characterization of microsatellite loci in caribou Rangifer tarandus, and their use in other artiodactyls. Mol. Ecol. 6, 697–699. 10.1046/j.1365-294X.1997.00237.x 9226951

[B51] Zerbino D. R. Birney E. (2008). Velvet: algorithms for de novo short read assembly using de Bruijn graphs. Genome Res. 18, 821–829. 10.1101/gr.074492.107 18349386 PMC2336801

