# Supplementary material for: Characterization of enteropathogenic and Shiga toxin-producing Escherichia coli in cattle and deer in a shared agroecosystem
Source: Front Cell Infect Microbiol. 2015 Apr 1;5:29. doi: 10.3389/fcimb.2015.00029 (PMC4381715; doi:10.3389/fcimb.2015.00029)

**Table S1**. Primer sequences for O-antigen polymerase (*wzy*).

| Serotype | Assay | Primers | Oligonucleotide sequences (5’-3’) | Amplicon |
|---|---|---|---|---|
| O2 | III | wzy2_F474 | TTGGTCTGCTTTGCCTTTTT | 111 bp |
| | | wzy2_R584 | TCTCCTGAGATCGCAATCAA | |
| O6 | III | wzy6_F370 | TATCCGCATGGTCCTTATCC | 299 bp |
| | | wzy6_R668 | CCCTTCAAGGTGCTAAATGC | |
| O26 | I | wzy26_F223 | TTAGGCGGTACCCATGAAGTCA | 242 bp |
| | | wzy26_R464 | GGTGCCATAAAGACAAAACAAAGA | |
| O45 | I | wzy45_F188 | ATCGCGTTCGTCTGGATGAAAT | 443 bp |
| | | wzy45_R630 | AGCGCCCCTGATATCTCCTACAG | |
| O53 | III | wzy53_F481 | CCATTTCAAACAAGTGAAGAAGG | 201 bp |
| | | wzy53_R681 | TGCCAACATCCCGTTTCTAT | |
| O91 | III | wzy91_F812 | AGTCGGGGCTTGTTTTTAGG | 384 bp |
| | | wzy91_R1195 | CAGATATGCCGACCGTTTTC | |
| O98 | III | wzy98_F123 | TGAACCAATACGCGGTTTTT | 1025 bp |
| | | wzy98_R1147 | ATGTGCTGTTGTGCTTGGAG | |
| O103 | II | wzy103_F929 | CCCCGCGGGTATTTGCTAT | 184 bp |
| | | wzy103_R1112 | TCGTATGCGTTCGTTCTAAGATAA | |
| O104 | -- | wzy104_F433 | TTAGCCGTTGCCATATTTTTGTAT | 429 bp |
| | | wzy104_R861 | ATTACCCGGGGCAATTATCATTA | |
| O111 | II | wzy111_F495 | TTCCGTAATTTGCATCCTGATAC | 549 bp |
| | | wzy111_R1043 | TTTGCAAATCCATAAACAACTCC | |
| O115 | III | wzy115_F113 | CATTCGTCGTGATGTGCATT | 690 bp |
| | | wzy115_R802 | TATTTGTCATCACCGCTTGC | |
| O121 | I | wzy121_F306 | TACAGCCGGTAGTGTTGAAAGGAT | 626 bp |
| | | wzy121_R931 | CGCCCGTGTTAATATTCCAAGTC | |
| O145 | I | wzy145_F754 | ATGGGCAGTATCTCTGGTATTGAA | 334 bp |
| | | wzy145_R1087 | TTGAAAGCCCGGATATTAGGAA | |
| O157 | II | wzy157_F586 | GTAGGGGTTGTATGCTCGTTGTT | 278 bp |
| | | wzy157_R863 | GCTCCCATGTCTCCAAATACTTGT | |
| O168 | III | wzy168_F285 | TTTTAGCGTTGCTGCTATAGTGA | 926 bp |
| | | wzy168_R1210 | CGAACCGTATAGCGGAAAAA | |
| O177 | III | wzy177_F442 | GGATTGATGGGGTCTGGTTA | 523 bp |
| | | wzy177_R964 | AGCCAGTACCCAGAACAGGA | |

**Table S2**. Primer sequences for flagellar H antigen (*fliC*) identification.

| Serotype | Assay | Primers | Oligonucleotide sequences (5'-3') | Amplicon |
|---|---|---|---|---|
| H2 | II | fliC2_F806 | TAGCGGGTACCCAGCCTCAAG | 305 bp |
| | | fliC2_R1111 | CATCGCTTCCACCCAGTTCAA | |
| H4 | II | fliC4_F544 | AGCGCGGCGAAACTGAC | 215 bp |
| | | fliC4_R760 | ACTTCCGCTGCACCAACA | |
| H7 | I | fliC7_F581 | CTGGCGCGAAGTTAAACACCA | 670 bp |
| | | fliC7_R1251 | ACCCGCGGTAAACCCAATAGTT | |
| H8 | I | fliC8_F633 | CGGCGCGGTTAAGAATGATG | 196 bp |
| | | fliC8_R829 | GCTCTGCGCCAGTGTTGTTAGTAA | |
| H10 | III | flic10_F272 | GTGTGCGTGAGCTGACTGTT | 555 bp |
| | | flic10_R826 | TGGCTTTAAGTGCAGAAGCA | |
| H11 | I | fliC11_F752 | CAGCGGCTAAGAATAAAGCACAGA | 237 bp |
| | | fliC11_R989 | GTGGCAGCCTTGTTATCAACTTTG | |
| H16 | III | flic16_F705 | TCCTTACCACCCACCTGAAC | 403 bp |
| | | flic16_R1107 | CCATTGAGATTGCCCTTGAT | |
| H19 | II | fliC19_F650 | CCGCGACTGCAAGCAATGTA | 493 bp |
| | | fliC19_R1143 | AGCCGCGTCTTTTAACACCTGA | |
| H21 | II | flic21_F430 | CGTGCTTCCTGTTTTCTTGG | 172 bp |
| | | flic21_R601 | TGAATTCACTATTTCGGGGAGT | |
| H25 | III | flic25_F579 | TGGCGATAAACCTGTTGATGT | 759 bp |
| | | flic25_R1337 | GGCCATTAGCCTTGTAACAG | |
| H28 | I | fliC28_F1040 | ACTGGCATACAACAGGCACACC | 387 bp |
| | | fliC28_R1427 | TTACCATCCGCTGAAACATAGACTG | |
| H34 | II | flic34_F423 | CGGTTCGATGAAAATTCAGG | 727 bp |
| | | flic34_1149 | AATGCCCGTAAATGCAGATG | |
| H38 | III | flic38_F916 | GCCGCCTTGAAGAATAACAC | 172 bp |
| | | flic38_R1087 | GCAGAGTCAGTGGATCGTTG | |
| H45 | III | flic45_F531 | CAAAGGCACTATTGCGAACA | 272 bp |
| | | flic45_R802 | CAGCCGCTGGTTTCAGAGT | |

**Figure S1**. Repetitive (rep)-PCR fingerprint profiles for a subset of deer and cattle diarrheagenic *E. coli* isolates. The Dice coefficient and a 0.5 % band position tolerance were used to identify isolates that were identical within and between animals. Cluster analysis was performed using the unweighted pair group method.

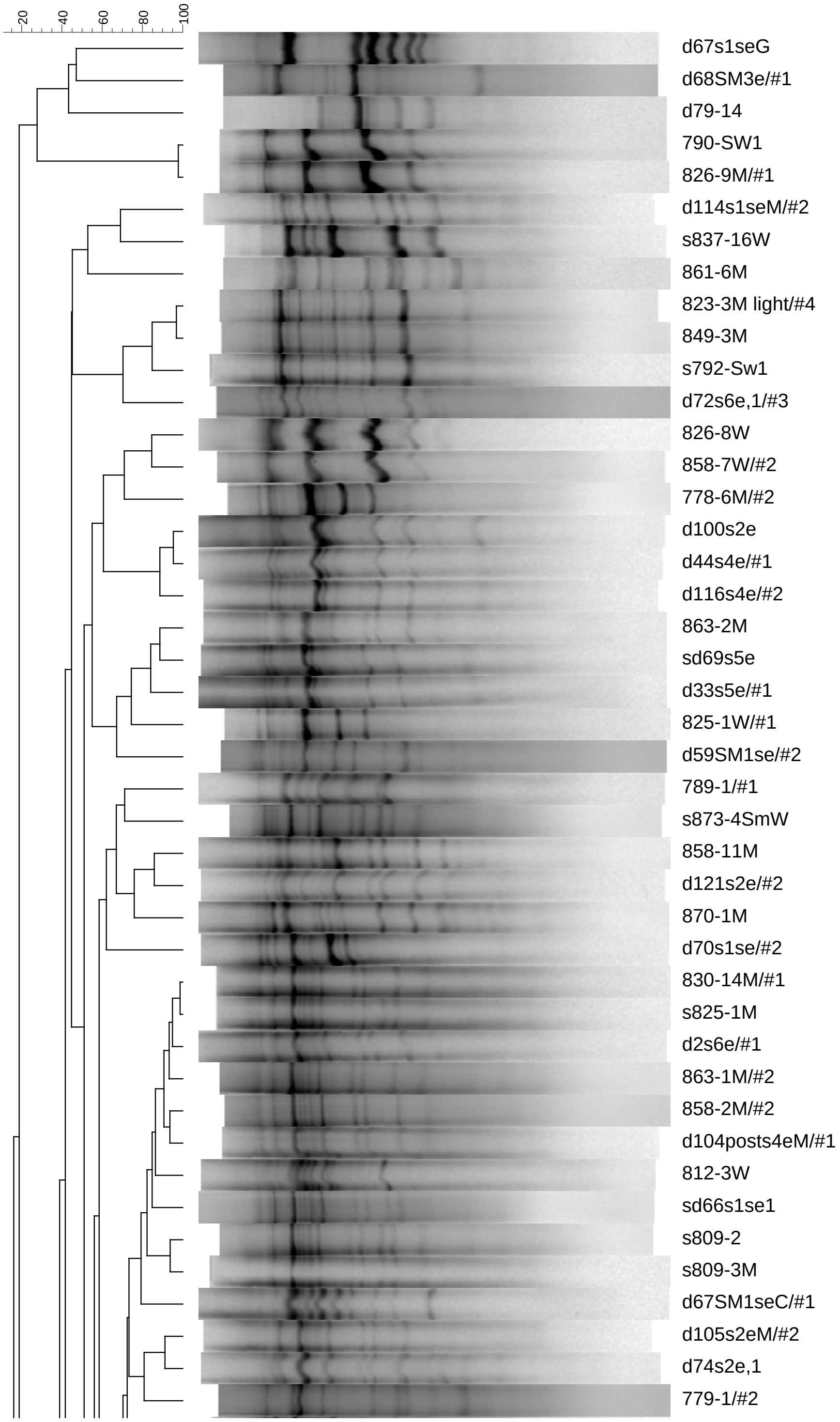

**Figure S1**., continued

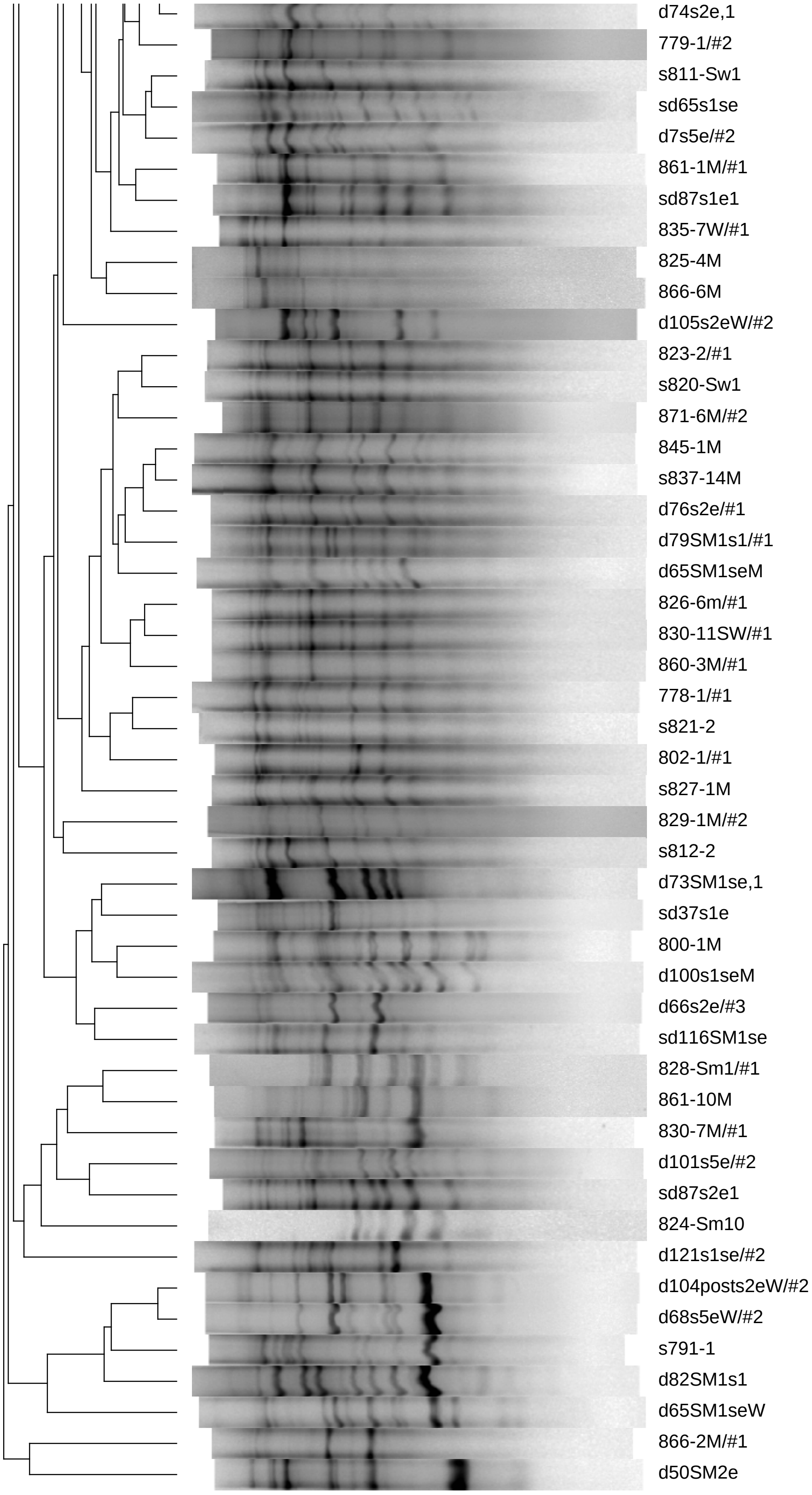

Supplement: Supplementary file 1 [file Presentation1.PDF]
